# *Clinical Clu‐Dr*: A Scalable Gamified Tool for Clinical Reasoning Practice

**DOI:** 10.1111/tct.70388

**Published:** 2026-03-05

**Authors:** Richard D. Horniblow, Linda Lefièvre, Louise Hammersley, Jacqueline Morgan, Lucas Tselepis, Rosanna Ghinai, Joseph Wheeler, Dawn Jackson

**Affiliations:** ^1^ Department of Biomedical Sciences, School of Infection, Inflammation, and Immunology, College of Medicine and Health University of Birmingham Birmingham UK; ^2^ Birmingham Medical School, College of Medicine and Health University of Birmingham Birmingham UK; ^3^ New Cross Hospital The Royal Wolverhampton NHS Trust Wolverhampton UK

## Abstract

**Background:**

Early development of clinical reasoning (CR) is essential but difficult to embed within preclinical medical curricula. Limited clinical exposure can hinder integration of basic and clinical sciences, whereas tutor‐led question‐and‐answer formats may limit engagement, and case‐based approaches often rely on clinical facilitators. *Clinical Clu‐Dr* (CC) was developed as a low‐resource, gamified approach to support early CR practice in a scalable format deliverable by both clinical and non‐clinical educators.

**Methods:**

Adapting the *Cluedo* concept, CC presents learners with a patient, potential diagnoses and 14–16 mixed‐format ‘*clues*’ (e.g., lab results, imaging and lifestyle data). In small groups, students analyse and share clues, construct differential diagnoses and confirm a final diagnosis. The design draws on scaffolded learning, cognitive integration and gamification.

**Evaluation:**

Evaluation combined post‐module student surveys (*n* = 128) with reflective facilitator feedback (*n* = 6; clinical/non‐clinical) collected across the delivery of 78 sessions. Surveys captured perceptions of engagement, confidence and clinical integration of the foundational biomedical science teaching, whereas facilitator reflections explored feasibility and delivery. Students reported higher perceived engagement and confidence. Facilitators noted initial challenges adapting to a student‐led format but reported strong, inclusive engagement.

**Implications:**

CC provides a scalable approach for embedding opportunities for CR practice in preclinical education. Although findings are based on self‐reported perceptions, the approach supports engagement and confidence. Successful implementation depends on careful alignment of clues with the module's basic science content and close collaboration between clinical and non‐clinical educators. Clinical expertise was not required for effective delivery.

## 1 Background

**FIGURE 1:**
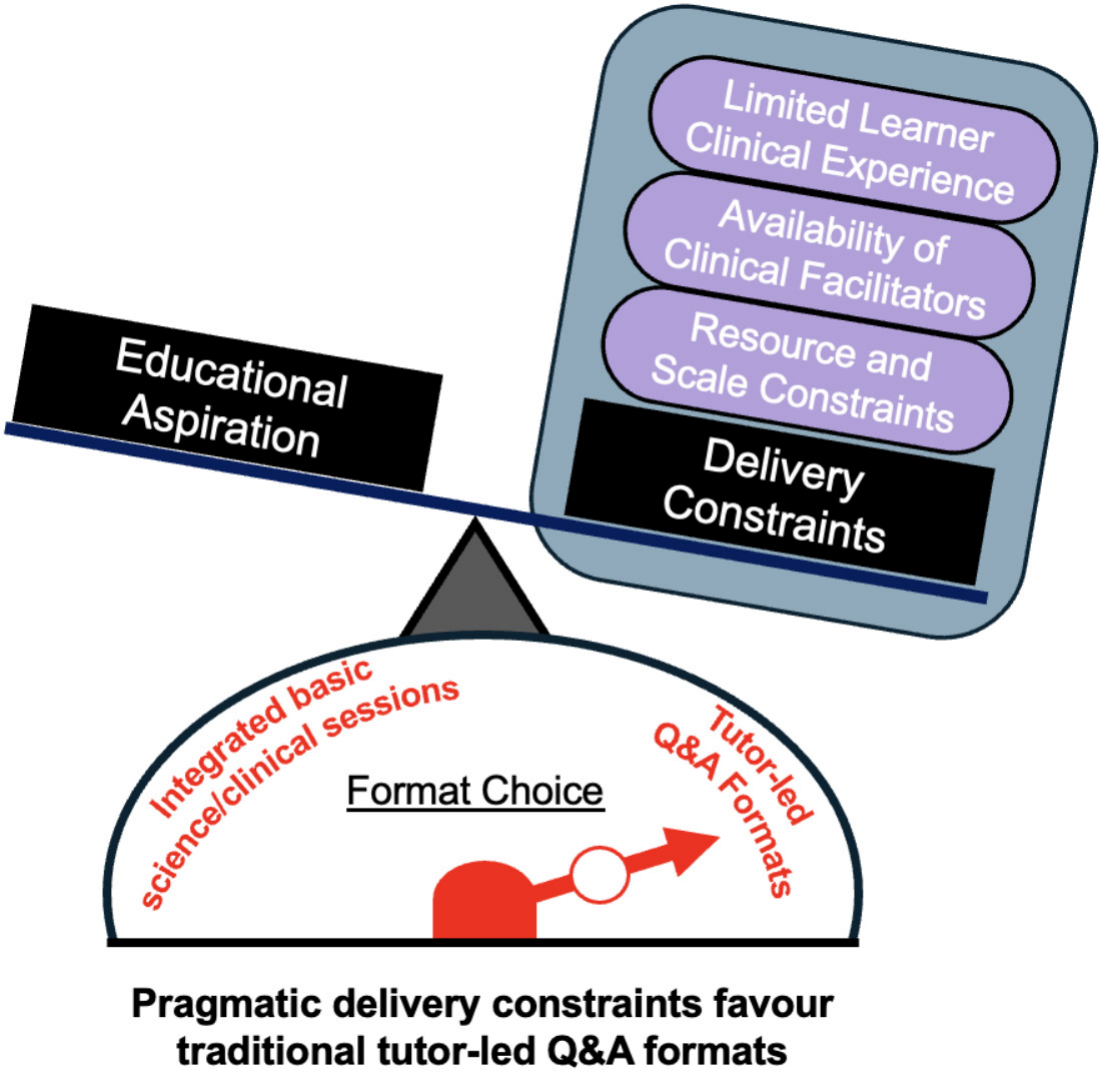
**Constraints shaping format choice in preclinical teaching**. Although integration of basic biomedical scientific content and CR is an educational aspiration, pragmatic delivery constraints often favour tutor‐led Q&A formats, limiting opportunities for explicit CR practice.

Clinical reasoning (CR) is a core capability underpinning safe and effective patient care. International recommendations, including those from the Clinical Reasoning in Medical Education (CReME) group, emphasise embedding CR early and strengthening integration between basic and clinical sciences [1]. However, the effective delivery of CR in early preclinical curricula is constrained by limited learner clinical experience, availability of clinical facilitators and resource and scale constraints [2], often resulting in tutor‐led *question‐and‐answer* (Q&A) formats with limited explicit focus on CR (Figure 1).

Similarly, at our institution, preclinical small‐group teaching (SGT) was predominantly delivered by basic science faculty, with limited opportunities for structured CR practice. Conventional, tutor‐led Q&A formats supported factual learning but offered fewer opportunities for learners to apply the foundational basic biomedical science to clinical contexts. This is a common challenge with traditional *knowledge‐first* approaches, where basic science is mastered before clinical exposure, particularly in large cohorts and resource‐limited settings [3, 4]. Real‐world clinical exposure is invaluable, but its intensity and pace in early training can limit opportunities for structured CR practice [5].

This prompted the redesign of these conventional Q&A SGT formats to create an approach that

1. Builds student confidence in reasoning despite limited clinical exposure.
2. Strengthens integration of foundational basic science with authentic clinical data.
3. Can be delivered effectively by both clinical and non‐clinical facilitators.
4. Requires minimal preparation and resources to enable scalability.

## 2 Approach

### 2.1 Innovation Design

**FIGURE 2:**
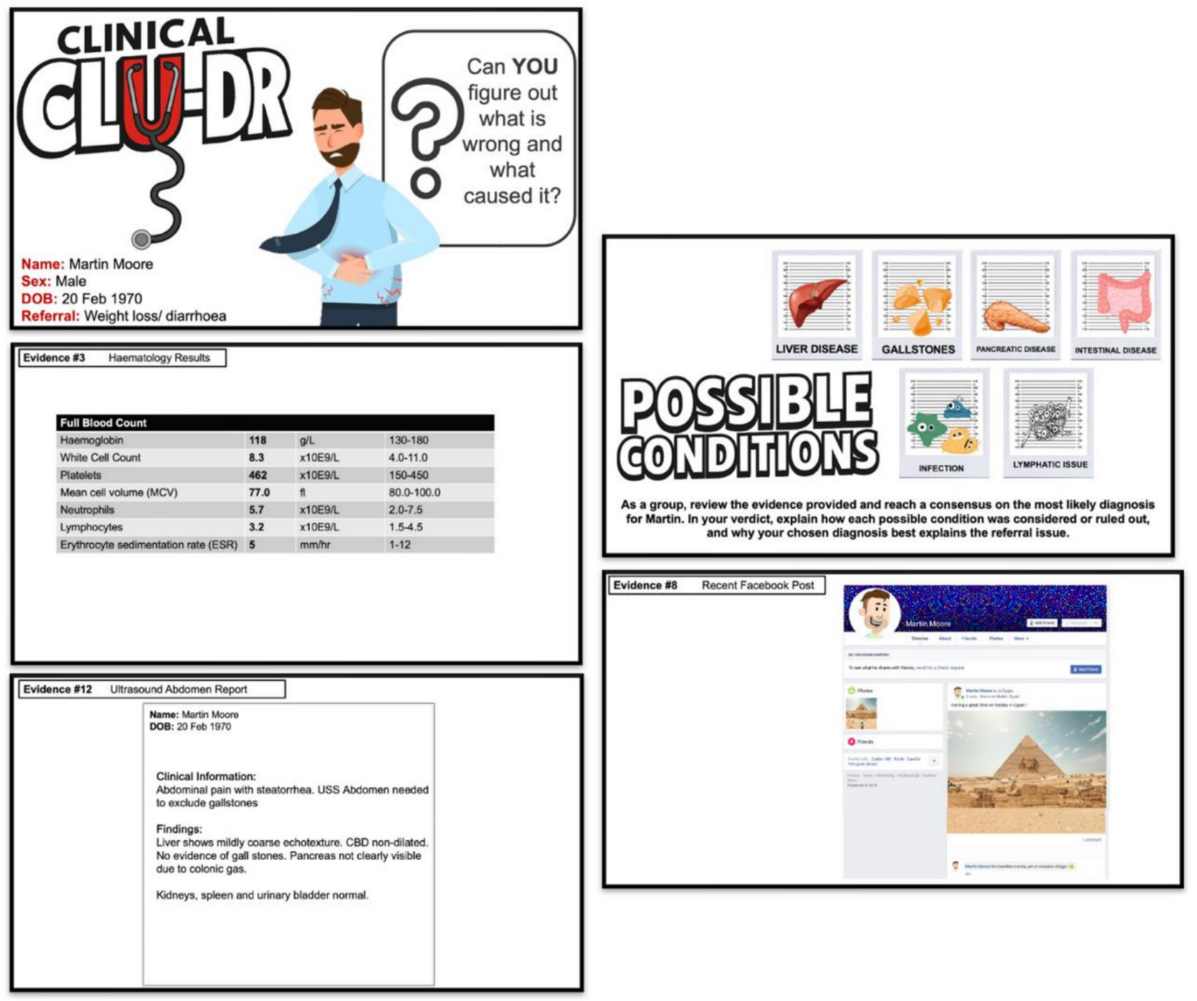
Example clues from a CC scenario.

*Clinical Clu‐Dr* (CC), which adapts the *Cluedo* concept, presents students with a patient, potential diagnoses and 14–16 ‘*clues*’. The evidence provided was mixed, with some clues more clinically focused (e.g., blood tests, imaging reports and prescriptions), whereas others were more anecdotal and lifestyle related (e.g., travel history and dietary habits); example clues are provided (Figure 2).

Each scenario followed a common template in which mixed‐format clues sequentially provided the elements of a clinical history, examination and investigation (reflecting the true process of clinical/diagnostic reasoning in practice) to scaffold learning [6], manage cognitive load and support holistic, stepwise CR.

These scenarios were developed by the module leads in collaboration with the Director of Education, with direct input from a clinician in medical education and advisory oversight from the College CR theme lead. Scenarios were aligned to module learning outcomes and prior tutor‐led Q&A topics, reviewed for clinical plausibility and appropriate Year 1 difficulty and designed with increasing complexity across the curriculum. Small‐group discussion and shared interpretation of clues were intentionally used to support learner‐centred and constructivist knowledge building [7], whereas the integration of authentic clinical data supported cognitive integration between basic and clinical sciences [4, 8]. Gamification and scaffolded learning can enhance engagement and make reasoning processes explicit; accordingly, light gamification elements were incorporated to support motivation and participation [9, 10].

### 2.2 Implementation

**FIGURE 3:**
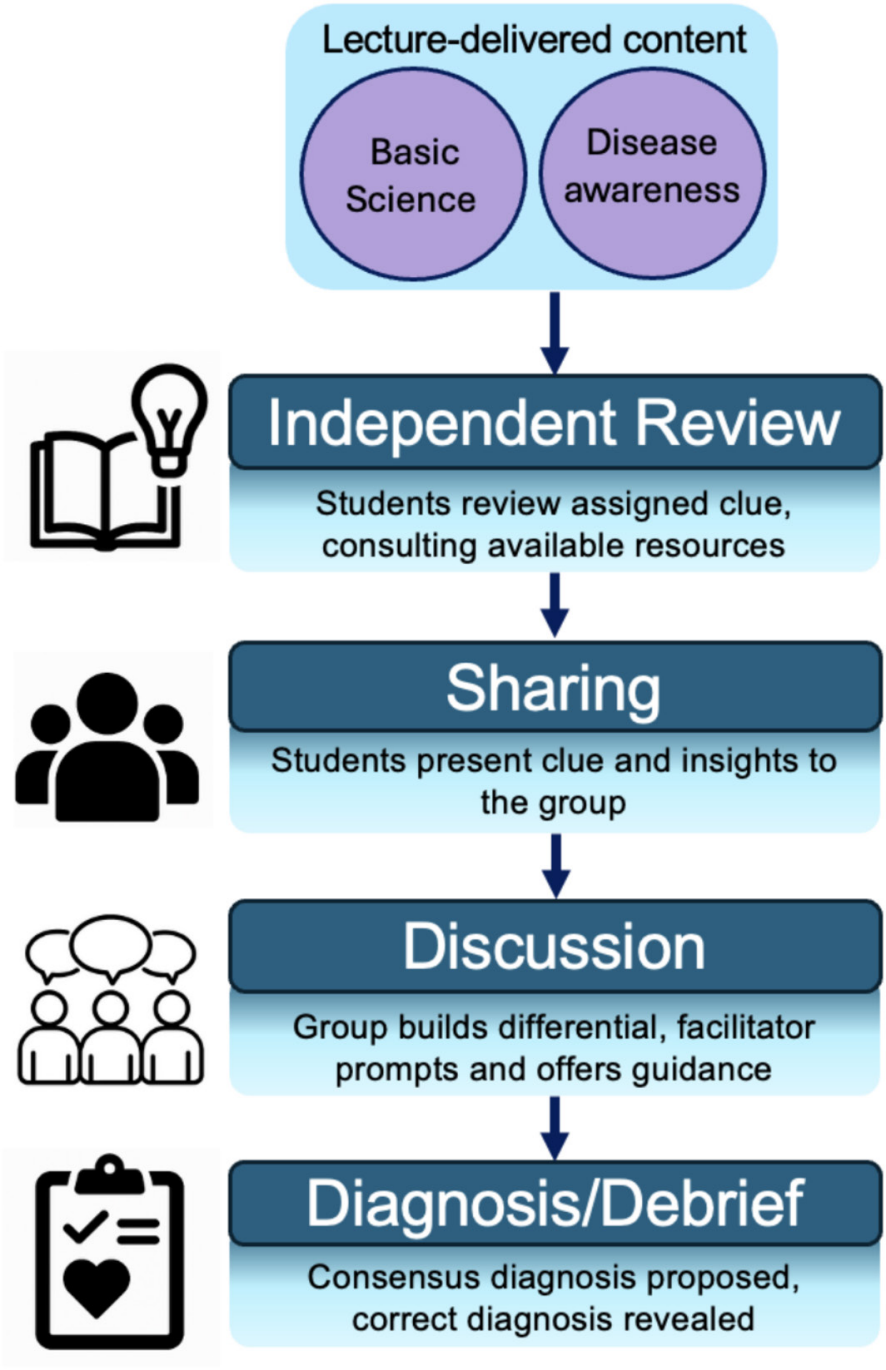
Structure and flow of a Clinical Clu‐Dr session.

CC was delivered to the full Year 1 preclinical cohort (*n* = 418) in 50‐min small groups (14–16 students, one tutor), with tutor–group assignments randomised; Figure 3 outlines the session format. Tutors were provided with brief facilitation guidance aligned to this format, alongside a tutor guide detailing the purpose of each clue and its links to underlying foundational basic science content.

Three sessions, aligned to Digestion‐Module topics, replaced the conventional Q&A SGTs previously delivered in that module, whereas other modules in the semester retained the conventional format; scenarios were built around the same topics previously addressed in Q&A sessions.

## 3 Evaluation

Recognising two key innovative elements (the implementation of the CC tool and the early incorporation of CR practice within the preclinical curriculum), this study evaluated student and facilitator experiences, guided by the following aims:

1. To explore student engagement, confidence and perceived learning experience in CC sessions compared with conventional SGT sessions.
2. To examine clinical and non‐clinical facilitator experiences of implementing CC, including feasibility and delivery.

The evaluation was designed as an early, exploratory assessment focusing on learner engagement, perceived relevance and facilitator experience, to establish the acceptability and feasibility of the innovation prior to a more in‐depth evaluation.

A triangulated evaluation combined an anonymous, optional post‐module student survey (*n* = 128) with reflective feedback from facilitators (three clinical teaching fellows and three non‐clinical educators) collected at the end of the module (survey questions are provided in Supporting Information). Students were asked to compare their experience of CC with conventional Q&A‐style SGT sessions (defined within the survey and experienced in the previous semester and concurrent teaching), allowing perceived differences in engagement and confidence to be quantitatively explored.

Reflective feedback from clinical and non‐clinical facilitators focused on the feasibility, benefits and challenges of delivery and was collected using guided prompts and informal discussion at the end of the module. Reflections were analysed using a framework analysis approach appropriate for applied educational evaluation [11]. Data were charted into a framework matrix, enabling comparison across clinical and non‐clinical facilitator perspectives and supporting interpretation of feasibility and implementation considerations. Representative comments were selected to illustrate key themes and to contextualise quantitative survey findings.

### 3.1 Mapping to Aims

**FIGURE 4:**
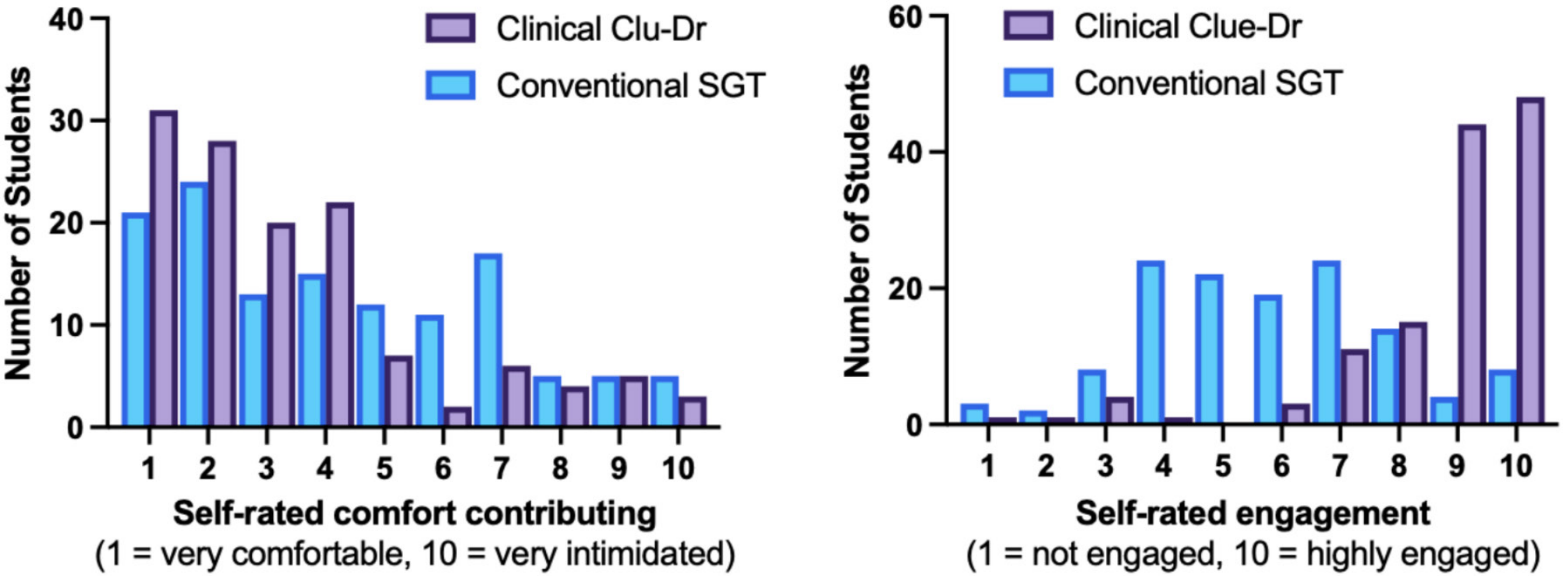
CC improves student confidence to participate and engagement compared to conventional SGT Q&A sessions.

Students reported greater confidence to contribute within CC sessions, with self‐rated comfort contributing in CC sessions higher than in a conventional SGT format (Figure 4).

**FIGURE 5:**
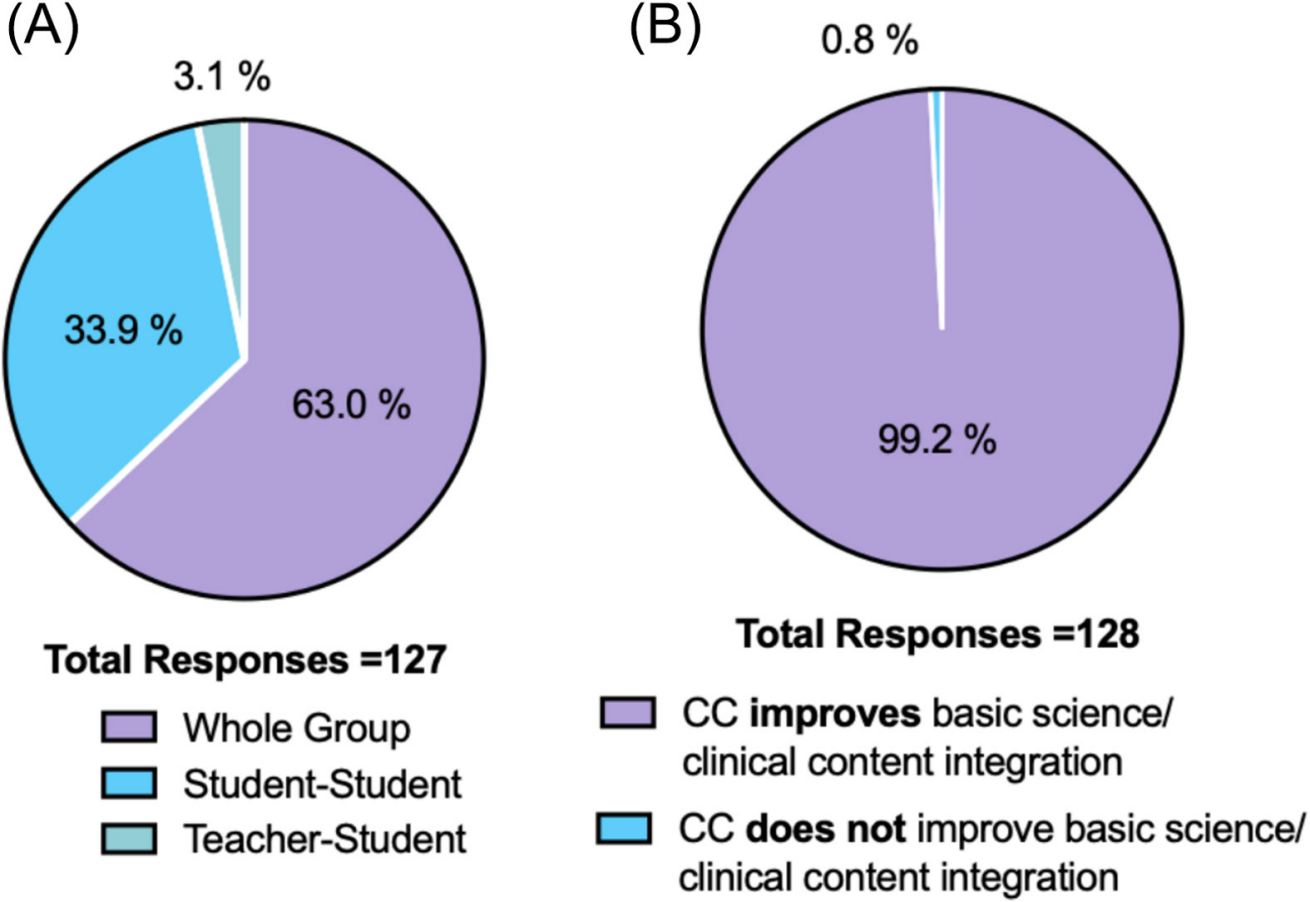
(A) Student perceptions of session focus and (B) student perceptions of integration of basic science and clinical content.

Students described the format as highly student‐focused, with collaborative interactions dominating the session (Figure 5A). Engagement ratings were also higher, and 98.4% valued the opportunity to practise CR skills. Almost all students (99.2%) agreed that CC improved their ability to integrate the foundational core scientific content with clinical contexts (Figure 5B).

Facilitators from both clinical and basic science backgrounds delivered sessions effectively; clinical facilitators confirmed that although specialist clinical expertise added contextual depth, it was not essential for effective facilitation of the session format, including supporting student engagement and structured discussion. Reflections highlighted strong participation and collaboration among students, with sessions fostering peer‐to‐peer discussion and whole‐group engagement. Non‐clinical facilitators described initial discomfort related to moving away from familiar tutor‐led, content‐reinforcing formats towards a more student‐led discussion style. This included uncertainty around reduced content coverage and managing open‐ended discussions. With experience across successive sessions, facilitators reported increased comfort as they observed high levels of inclusive participation and sustained peer‐to‐peer discussion.

*Clinical facilitators confirmed that although specialist clinical expertise added contextual depth, it was not essential for effective facilitation of the session format, including supporting student engagement and structured discussion*.

These gamified activity sessions required a different approach … they were designed to be student‐led, which was outside my comfort zone. (Non‐clinical facilitator feedback excerpt)

Being a clinician was not required … but having a clinical background gave a different perspective, allowing us to introduce students to the basic tenets of clinical information gathering and reasoning. (Clinical facilitator feedback excerpt)

Taken together, student survey findings and facilitator reflections support the stated aims of the innovation, demonstrating high perceived engagement, opportunities to practise early analytic, hypothesis‐driven reasoning processes and feasibility across both clinical and non‐clinical facilitation contexts.

## 4 Implications

CC is a gamified, clue‐based, scaffolded case discussion format that promotes the integration of foundational basic science content with clinical data and reasoning practice in preclinical teaching. It is low‐resource, deliverable by both clinical and non‐clinical facilitators, and aligns well with current recommended educational frameworks for CR [1].

*CC is a gamified, clue‐based, scaffolded case discussion format that promotes the integration of foundational basic science content with clinical data and reasoning practice in preclinical teaching*.

Key lessons from implementation highlight the importance of careful scenario and clue delivery. Effective delivery depended on constructing a clue base grounded in taught basic science content, enabling students to relate clinical evidence to prior learning while supporting facilitator transition through familiar topics from previous SGT. Close collaboration between clinical and non‐clinical educators during the design phase was essential to ensure clinical plausibility while maintaining accessibility for non‐clinical facilitation. Although some non‐clinical facilitators were initially hesitant (reflecting a shift away from familiar tutor‐led, content‐reinforcing formats and concerns about not revisiting lecture content, the fluidity of the session and its student‐led nature), clear facilitation guidance and structured session support helped them recognise benefits for student engagement and their own facilitation practice. The scaffolded structure allowed facilitators to progressively step back as learners grew in confidence [6, 12], supporting the transition from novice to a more independent clinical thinker.

Several limitations should be considered. As an early, perception‐focused evaluation, this study does not assess higher‐order learning or behavioural outcomes, which should be examined in future work (e.g., using CR task checklists [13] or empirically derived scales [14]). Evaluation outcomes were based on self‐reported perceptions rather than objective measures of CR performance, and increased engagement may partly reflect novelty associated with the format. Implementation experience also highlighted variability in group dynamics and the need to carefully calibrate clue complexity, as some evidence required differing depths of exploration and less detailed clues were sometimes initially undervalued, reinforcing the importance of structured whole‐group synthesis to support shared learning. Practical refinements identified include clearer guidance on when facilitators should intervene during reasoning discussions, improved consistency through enhanced briefing materials and optional post‐session consolidation opportunities that support learning without reverting to tutor‐led delivery.

Successful implementation required close collaboration between clinical and non‐clinical educators and alignment with existing module content. Facilitator adoption was supported by continuity with prior tutor‐led Q&A formats, as scenarios were built around familiar conditions and question themes. Delivery was most effective in small‐group settings; attempts to run sessions in very large groups limited interaction. Although non‐clinical facilitators may be less able to draw on clinical anecdotes to illustrate real‐world practice, this limitation is balanced by the structured, low‐resource design, offering a complementary alternative to traditional case‐based approaches that typically require clinical facilitation and may become repetitive.

*Successful implementation required close collaboration between clinical and non‐clinical educators and alignment with existing module content*.

At our institution, this model will be expanded across other modules in preclinical medicine through a curriculum review process. Although anchored to preclinical teaching, the structure is transferable to other basic science contexts where scientific principles can be meaningfully linked to clinical decision‐making. Beyond embedding early CR practice, the longer‐term aim is to use shared clinical cases to transcend individual modules (e.g., linking immunology with digestion) and support a spiral curriculum that reinforces learning over time. Implementation experience highlighted several areas for further refinement. Clearer guidance for facilitators on when to intervene during reasoning discussions would better support groups that become uncertain or diverge substantially, while preserving the student‐led nature of the sessions. Greater consistency across facilitators may be achieved through enhanced briefing materials, and optional post‐session consolidation opportunities could support learning without reverting to tutor‐led delivery. These refinements will inform future iterations of the approach.

## Author Contributions

**Richard D. Horniblow:** conceptualization, investigation, methodology, formal analysis, data curation, project administration, visualization, writing – original draft, writing – review and editing. **Linda Lefièvre:** investigation, methodology, writing – original draft. **Louise Hammersley:** methodology, writing – original draft. **Jacqueline Morgan:** investigation, writing – original draft. **Lucas Tselepis:** investigation, writing – original draft. **Rosanna Ghinai:** investigation, writing – original draft. **Joseph Wheeler:** conceptualization, methodology, visualization, writing – original draft, writing – review and editing. **Dawn Jackson:** investigation, methodology, writing – original draft, writing – review and editing.

## Funding

The authors have nothing to report.

## Ethics Statement

This study was conducted according to the guidelines of the Declaration of Helsinki and approved by the University of Birmingham Science, Technology, Engineering and Mathematics Ethics Committee (ERN_2117, February 2024).

## Conflicts of Interest

The authors declare no conflicts of interest.

## Supporting information


**Data S1:** Supporting Information.

## Data Availability

All data and teaching resources are available upon request.
