# Supplementary material for: Clinical Clu‐Dr: A Scalable Gamified Tool for Clinical Reasoning Practice
Source: Clin Teach. 2026 Mar 5;23(2):e70388. doi: 10.1111/tct.70388 (PMC12962845; doi:10.1111/tct.70388)
Supplement: Supplementary file 1 — Data S1: Supporting Information. [file TCT-23-e70388-s001.docx]

**Supplementary Information**

Questionnaire used for Student Survey:

Thank you for taking part in the survey. Please be aware that by taking part in this survey and answering these questions, you are consenting to us using the data for research purposes. None of the questions and/or information you provide will identify you, and all answers will thus be anonymous.

**Question 1:** In the session, did you feel/envision yourself in the role as a team of doctors/clinicians discussing a real-life clinical scenario?

Yes/ No

**Question 2:** Do you think the format of Clinical Clu-Dr sessions improves your appreciation and integration of the physiology you are taught in the main lecture sessions to clinical concepts?

Yes/ No

**Question 3:** On a scale of 1-10, how uncomfortable/intimidated do you feel contributing in a conventional SGT?

1 = not uncomfortable at all…to…10 = very uncomfortable

**Question 4:** On a scale of 1-10, how uncomfortable/intimidated do you feel contributing to the clinical aspects of your clue in these Clinical Clu-Dr sessions*?*

1 = not uncomfortable at all…to…10 = very uncomfortable

**Question 5:** On a scale of 1-10, how engaged do you feel in a conventional SGT? session.

1= not engaged at all…to…10 – highly engaged

**Question 6:** On a scale of 1-10, how engaged are you in these Clinical Clu-Dr?

1= not engaged at all…to…10 – highly engaged

**Question 7:** How would you describe the Clinical Clu-Dr sessions: teacher-focused or student-focused?

Teacher Focussed/ Student Focussed

**Question 8:** Please select which of the following interactions describes the session best:

- 1. Predominantly, interactions between the facilitator/tutee and individual learners.
  2. Multiple, active interaction between the facilitator/tutee, individual learners, and their peers.
  3. Predominantly, interactions amongst the individual learners/students

***Question 9:*** When comparing these Clinical Clu-Dr sessions to a conventional SGT, do you think the format improves the way you share your ideas and attitudes with peers/students in the group?

**Note:** a conventional SGT session refers to a session where you are provided a set of questions prior to the session, and the answers are provided and explained in the session by the tutor.

**Question 10:** Do you know what clinical reasoning is?

Yes/No/Maybe-but unsure

**Definition:** *'Clinical reasoning'* describes the thinking and decision-making processes associated with clinical practice.

**Question 11:** Do you think that Clinical Clu-Dr sessions have enabled you to practice/learn these skills, in comparison to a conventional SGT?

Yes/ No/ Unsure as still don’t fully understand what clinical reasoning means

**Question 12:** Given you are a Y1 student, what do you feel the benefits of this session are over following similar scenarios, perhaps at the bedside, in the clinic?

**Question 13:** Are there any disadvantages?
